# Intensive care acquired infection is an independent risk factor for hospital mortality: a prospective cohort study

**DOI:** 10.1186/cc4902

**Published:** 2006-04-20

**Authors:** Pekka Ylipalosaari, Tero I Ala-Kokko, Jouko Laurila, Pasi Ohtonen, Hannu Syrjälä

**Affiliations:** 1 Department of Infection Control, Oulu University Hospital, FIN-90029 OYS, Finland; 2 Department of Anesthesiology, Division of Intensive Care, Oulu University Hospital, FIN-90029 OYS, Finland; 3 Departments of Anesthesiology and Surgery, Oulu University Hospital, FIN-90029 OYS, Finland

## Abstract

**Introduction:**

The aim of this study was to elucidate the impact of intensive care unit (ICU)-acquired infection on hospital mortality.

**Methods:**

Patients with a longer than 48 hour stay in a mixed 10 bed ICU in a tertiary-level teaching hospital were prospectively enrolled between May 2002 and June 2003. Risk factors for hospital mortality were analyzed with a logistic regression model.

**Results:**

Of 335 patients, 80 developed ICU-acquired infection. Among the patients with ICU-acquired infections, hospital mortality was always higher, regardless of whether or not the patients had had infection on admission (infection on admission group (IAG), 35.6% versus 17%, *p *= 0.008; and no-IAG, 25.7% versus 6.1%, *p *= 0.023). In IAG (*n *= 251), hospital stay was also longer in the presence of ICU-acquired infection (median 31 versus 16 days, *p *< 0.001), whereas in no-IAG (*n *= 84), hospital stay was almost identical with and without the presence of ICU-acquired infection (18 versus 17 days). In univariate analysis, the significant risk factors for hospital mortality were: Acute Physiology and Chronic Health Evaluation (APACHE) II score >20, sequential organ failure assessment (SOFA) score >8, ICU-acquired infection, age ≥ 65, community-acquired pneumonia, malignancy or immunosuppressive medication, and ICU length of stay >5 days. In multivariate logistic regression analysis, ICU-acquired infection remained an independent risk factor for hospital mortality after adjustment for APACHE II score and age (odds ratio (OR) 4.0 (95% confidence interval (CI): 2.0–7.9)) and SOFA score and age (OR 2.7 (95% CI: 2.9–7.6)).

**Conclusion:**

ICU-acquired infection was an independent risk factor for hospital mortality even after adjustment for the APACHE II or SOFA scores and age.

## Introduction

Patients admitted into intensive care units (ICUs) are at great risk for acquiring nosocomial infections. They are susceptible to infection because of their underlying diseases or conditions associated with impaired immunity as well as several violations of their immune system or risks of aseptic mistakes in patient management during invasive monitoring and they are prone to secondary infections after exposure to broad-spectrum antimicrobials [1].

Prevalence or prospective cohort studies have earlier shown ICU-acquired infections to be associated with high mortality, excessive length of ICU and hospital stay, and high hospital costs [2-5]. However, the significance of ICU-acquired infection for patient outcome is controversial. In one earlier case-control study, after adjustment for risk factors, ICU-acquired catheter-related infection was not a significant risk factor for mortality [6]. In other studies on catheter-related infections, the patients with infection had longer hospital stays than the controls, with no difference in mortality [7]. In studies based on large sets of register data [8] and a case-control design [9], ventilator-associated pneumonia (VAP) was associated with longer hospital stay but no effect on mortality. A recent meta-analysis of VAP, however, showed that the cases with VAP had a two fold mortality rate compared to matched controls [10]. Increased mortality has also been reported among ICU patients with Gram-negative bacteremia [11,12] or intra-abdominal infections [13].

We were interested in whether ICU-acquired infections had an impact on the outcome in our severely ill mixed medical-surgical ICU patient population. For this prospective analysis, we included each patient who stayed in our ICU for more than 48 hours during a 14 month study period.

## Materials and methods

### Study location and patients

This study was conducted at Oulu University Hospital, which is a 900-bed tertiary-level university hospital. The mixed medical-surgical ICU is a 10-bed unit with one 6-bed, one 2-bed, and two single-bed rooms. This ICU has 700 to 750 annual admissions, and 49% of the admissions are surgical, 41% medical and 10% from other specialties. All patients admitted into the ICU for more than 48 hours during the study period from May 2002 to June 2003 were included in the study. They were prospectively followed up until discharge from hospital or death. The Hospital Ethics Committee approved the study design. Because the study was epidemiological without any interventions the informed consent was waived.

### Study parameters

The following information was collected for all study patients: age, gender, cause of admission, severity of underlying diseases, and organ dysfunction on admission as assessed by means of the Acute Physiology and Chronic Health Evaluation (APACHE) II index [14] and the Sequential Organ Failure Assessment (SOFA) score [15], Glasgow Coma Scale, smoking habits, alcohol or drug abuse, presence of ischemic heart disease, chronic obstructive pulmonary disease, asthma, diabetes mellitus, chronic renal or hepatic failure, underlying malignancy, recent use of immunosuppressive therapy, elective or emergency operations during the preceding 14 days, infection on admission, and previous antimicrobial therapy. The intensity of treatment was recorded by the Therapeutic Intervention Scoring System (TISS) score [16].

Urine bacterial culture was routinely performed on admission. Microbiological samples of blood, urine, tracheobronchial secretions, and any suspected infection focus were always obtained when a new infection was suspected. The length of stay (LOS) in the ICU and at hospital were recorded, as were ICU and hospital deaths.

### Classification of infection

Infections present on admission into the ICU were considered community-acquired if they were already manifested on admission into hospital. An infection manifested >48 hours after admission was defined as hospital acquired. Infections that developed 48 hours after admission into the ICU were considered ICU acquired. The presence and criteria of infection were assessed daily on the ward round together with an infectious disease specialist and the ICU physicians.

The definitions of infections were based on the definitions proposed by the Centers for Disease Control and Prevention with the following modification [17,18]: a catheter-related infection was diagnosed when the same strains of bacteria were isolated in blood cultures and in semi-quantitative catheter tip cultures, when no other site of infection was present. A catheter-related infection was also diagnosed if the patient had a positive semi-quantitative catheter tip culture while blood cultures showed no growth or were not done, clinical signs of infection without other sites of infection, and a favorable response to antimicrobial therapy.

### Data registration and statistical analysis

Data were collected daily by one of the authors (PY) and entered into an SPSS database (SPSS Data Entry, version 2.0, SPSS Inc., Chicago, IL, USA). Summary statistics for continuous or ordinal variables were expressed as the median with 25th and 75th percentiles. The analyses of the differences between the infection and no-infection groups were performed by Student's *t *test or Mann-Whitney *U* test (the latter in the case of non-normally distributed data). Kruskal-Wallis test was used for continuous variables in comparisons of several groups. Categorical variables were analyzed by Pearson Chi-square test or Fisher's exact test. Predicted mortality with a 95% confidence interval (CI) was calculated according to the APACHE II risk score [14]. Logistic regression analysis was used to evaluate the odds ratios (ORs) with 95% CI. Two parallel multivariable models were built: an APACHE score and age-adjusted model; and a SOFA score and age-adjusted model. All potentially significant (*p *≤ 0.20) variables were entered into both models. Possible interactions between ICU-acquired infection and other variables in the final models were analyzed. The linearity assumption of continuous variables (APACHE II and SOFA scores and age) was checked by creating a design variable based on quartiles. Goodness-of-fit was evaluated by Hosmer-Lemeshow test. Two-tailed *p *values are reported, and the analyses were performed using SPSS software (version 12.0.1, SPSS Inc., Chicago, IL, USA).

## Results

### Characteristics of ICU admissions

The total number of patients admitted during the study period was 817, of whom 429 (52.5%) had an ICU LOS >48 hours. The study population has been described in more detail elsewhere [19]. Briefly, 94 patients were excluded: 27 patients with ICU readmissions, 23 patients due to incomplete data, and 44 patients with an ICU-acquired infection on admission, having been transferred from another ICU. Thus, the final study population comprised 335 patients; 23.9% (*n *= 80) of the patients developed a total of 107 ICU-acquired infections during their ICU stay. The following infections were seen in a descending order of frequency: VAP (*n *= 27), surgical site infections (21), lower respiratory tract infection (16), intra-abdominal infections (15), sinusitis (11), soft tissue or skin infections (6), primary or catheter-associated bacteremia (5), secondary bacteremia (4) and urinary tract infection (1).

Table 1 presents the age, sex, and severity scores of the patients. APACHE II scores did not differ between the groups (*p *= 0.87); however, the patients with ICU-acquired infection had higher median SOFA scores on admission than those without ICU-acquired infection (Table 1).

### Impact of ICU-acquired infection on hospital mortality

In univariate analysis, the significant risk factors for hospital mortality were SOFA score >8 on admission, APACHE II score >20, ICU-acquired infection, age ≥ 65 years, community-acquired pneumonia on admission, malignancy or immunosuppressive medication, and ICU stay >5 days (Table 2). In the multivariable analyses, the first model was adjusted by APACHE II score and age (Table 3) and the second model by SOFA score and age (Table 4). All potentially significant variables according to the univariate analysis were also entered in those models. After adjustment, ICU-acquired infection remained as a risk factor in both models. Immunosuppressive medication and community-acquired pneumonia were the most significant adjusting factors in the models adjusted for APACHE II score and age and SOFA score and age. No significant interactions were found between ICU-acquired infection and other variables in the final models.

### Outcome

Clinical outcome was analyzed in four groups: the groups having no infection or already having infection on admission and the corresponding groups with or without ICU-acquired infection (Table 5). Although ICU mortality did not differ significantly between the groups, ICU LOS was longer in the patients with ICU-acquired infection. On the other hand, among the patients who had acquired an ICU infection, hospital mortality was higher regardless of whether they had no infection (25.7% versus 6.1%, *p *= 0.023) or had an infection (35.6% versus17%, *p *= 0.008) on admission. In the whole study population, the ratio of observed to predicted mortality (calculated according to APACHE II score on admission) was 0.406 (95% CI 0.31–0.52), while in patients without ICU infection the ratio was clearly lower regardless of whether or not they had infection on admission (Table 5). Nor did hospital LOS differ among the patients with no infection on admission regardless of whether or not they acquired infection during their ICU stay. The situation was clearly different among the patients with infection on admission; ICU infection prolonged their hospital stay 2.2-fold (*p *< 0.001). Furthermore, ICU-acquired infection increased the TISS scores in both groups: they were 1.9-fold in the group with no infection on admission (*p *< 0.001) and 4.1 fold in the group with infection on admission (*p *< 0.001).

## Discussion

Our results show that ICU-acquired infection remained a significant risk factor for hospital mortality even after adjustment for the APACHE II and SOFA scores and age. ICU-acquired infection also increases resource use and the length of hospital treatment.

The impact of ICU infections on hospital mortality is controversial. Prevalence and prospective cohort studies have reported various ICU infections to be independent risk factors for hospital mortality, including pneumonias or bloodstream infections [2,3,20], or ICU infections as a whole [5]. Other studies have reported increased mortality without analysis of confounding factors [21,22]. In contrast, earlier case-control studies have failed to reveal any difference in mortality between patients with ICU infection and their controls [7,9]. Similarly, in a very recent study, ICU-acquired infection was not an independent risk factor for post-ICU in-hospital mortality [23]. Our results support the findings of ICU-acquired infections increasing hospital mortality: the attributable mortality from ICU-acquired infection was 19.6% in the patients without infection on admission and 18.6% in the patients infected on admission. The impact of ICU infection on hospital mortality was highest among the patients without infection on admission, whose observed/predicted mortality ratio was five fold compared to the patients without ICU infection, which is in harmony with the earlier literature [24].

The groups had different lengths of hospital stay. Among the patients with infection on admission, the excess length of hospital stay was 15 days. Surprisingly, ICU infection increased the hospital stay of the patients without infection on admission by only one day, which may reflect the fact that altogether 25.7% of the patients who acquired an ICU infection died, causing a shorter hospital stay. This is in contrast to an earlier report, where hospital LOS was increased in the presence of an ICU infection irrespective of a patient's infection status on admission [24]. Furthermore, in concordance with an earlier report [25], our patients with ICU-acquired infection needed significantly more resources based on the consumed TISS scores in both groups, which shows that ICU infections are expensive and laborious to treat.

The APACHE II score was initially developed for predicting the risk of death in an ICU population [14]. The relationship between ICU infection and mortality has earlier been reported to be modified by the APACHE II score: the highest influence of nosocomial infection on mortality rate was observed for APACHE II scores of 11 to 30, because patients with high APACHE II scores may die from their underlying disease before they develop an infection [5]. In our series, admission APACHE II scores did not differ between the groups with and without ICU infection. In APACHE and age-adjusted multivariate analysis, ICU-acquired infection remained an independent risk factor for hospital mortality.

The SOFA score was developed to assess organ dysfunction *per se *independently of the underlying disease [15]. It was noted earlier that a greater degree of organ dysfunction on admission or during the ICU stay was related to subsequent infection during intensive care [21]. Similarly, in our series, SOFA scores were higher on admission among the patients who later developed an ICU-acquired infection. It has also been reported that, in addition to the severity scores recorded on admission, daily increase of the illness severity score within the first four days post-admission was associated with an increased risk of death in the ICU [26]. Even after adjustment for these severity scores, however, late-onset VAP was associated with an increased risk of death. In one case-control study, ICU-acquired catheter-related septicemia was associated with significant attributable mortality after adjustment only for admission severity scores, whereas after adjustment for severity scores at 3 or 7 days before the onset of nosocomial bacteremia, there was only a trend toward catheter-related septicemia-attributable mortality [6]. Our multivariate analysis showed that an ICU-acquired infection remained an independent risk factor for hospital mortality even after adjustment for SOFA score and age. Successive SOFA scores were not available for adjustment in our series.

While infection on admission in general was not a risk factor for hospital mortality in univariate analysis, community-acquired pneumonia was clearly associated with increased mortality. In an earlier retrospective study, community-acquired pneumonia requiring mechanical ventilation was not associated with higher mortality compared to non-community-acquired pneumonia ICU patients [27]. In harmony with the earlier literature, immunosuppressive medication and malignancy were independent risk factors for hospital mortality in our ICU population [20,28,29].

Our aim was to evaluate the impact of ICU infections in general on hospital mortality, for which controversial results have been reported earlier. General evaluation is also important for administrative purposes and ICU planning. Although the analysis of specific infections was outside the scope of our interest in this study, univariate analysis showed that the OR of VAP to hospital mortality (OR = 2.5; 95% CI 1.06–5.91) was not higher than the OR of ICU infections as a whole (OR = 2.6; 95% CI 1.45–4.66).

## Conclusion

ICU-acquired infection was an independent risk factor of death during the hospital stay even after adjustment for different underlying conditions. It also increased resource use and the length of hospital treatment. The impact on hospital mortality was highest in the patients without infection on admission.

## Key messages

• ICU-acquired infection was an independent risk factor for hospital mortality even after adjustment for APACHE II or SOFA scores and age.

• The impact of an ICU infection on hospital mortality was highest among the patients without infection on ICU admission.

• ICU-acquired infection increased resource use and the length of hospital treatment among the patients with infection on admission.

## Abbreviations

APACHE = Acute Physiology and Chronic Health Evaluation; CI = confidence interval; ICU = intensive care unit; LOS = length of stay; OR = odds ratio; SOFA = Sequential Organ Failure Assessment; TISS = Therapeutic Intensity Scoring System; VAP = ventilator-associated pneumonia.

## Competing interests

The authors declare that they have no competing interests.

## Authors' contributions

PY participated in the design of the study and acquisition and analysis of data, and drafted the manuscript. TA-K, JL, and HS participated in the design of the study and the analysis of data and drafted the manuscript. PO participated in the design of the study and performed the statistical analysis. All authors read and approved the final manuscript.

## Figures and Tables

**Table 1:** Baseline demographic and clinical characteristics of patients

Characteristic	No ICU-acquired infection (*N *= 255)	ICU-acquired infection (*N *= 80)	*P *value
Male sex	159 (62.4)	58 (72.5)	0.11
Age (years)	59 (47–70)	59.5 (47–69)	0.70
APACHE II score on admission	23 (18–28)	22 (18–29)	0.87
SOFA score on admission	6 (4–9)	9 (6.8–10)	<0.001

Values are presented as median (with 25th to 75th percentile in parentheses) or as the number (percentage) of patients. APACHE, Acute Physiology and Chronic Health Evaluation; ICU, intensive care unit; SOFA, Sequential Organ Failure Assessment.

**Table 2:** Risk factors for hospital mortality: univariate analysis

Risk factor	Odds ratio	95% CI	*P *value
Age ≥ 65 years	2.28	1.3–3.93	0.004
Female	0.76	0.42–1.36	0.35
APACHE II >20	2.90	1.51–5.59	0.001
SOFA ≥ 8	4.28	2.24–8.17	<0.001
ICU-acquired infection	2.6	1.45–4.66	0.001
Immunosuppressive medication or malignancy	2.56	1.45–4.51	0.001
Diabetes	1.85	1.0–3.43	0.052
History of stroke or TIA	1.58	0.78–3.18	0.2
Current smoker	0.95	0.52–1.76	0.88
Alcohol abuse	0.59	0.27–1.32	0.2
Infection on admission	1.53	0.77–3.03	0.22
Community-acquired infection	1.37	0.79–2.37	0.26
Community-acquired pneumonia	2.3	1.29–4.06	0.005
Hospital-acquired infection	0.98	0.54–1.79	>0.9
Hospital-acquired pneumonia	1.0	0.46–2.18	>0.9
Operation <14 days	0.59	0.32–1.07	0.084
ICU LOS >5 days	1.91	1.10–3.34	0.022

APACHE, Acute Physiology and Chronic Health Evaluation; CI, confidence interval; ICU, intensive care unit; LOS, length of stay; SOFA, Sequential Organ Failure Assessment; TIA, transient ischemic attack.

**Table 3:** APACHE II and age-adjusted multivariate analysis of risk factors for hospital mortality

	OR	95% CI	*P *value
ICU-acquired infection	4.0	1.99–7.88	< 0.001
Malignancy or immunosuppressive medication	2.3	1.24–4.46	0.009
Community-acquired pneumonia	4.1	2.02–8.13	<0.001

-2 Log likelihood 264.766, P (Hosmer and Lemeshow test) = 0.548. APACHE, Acute Physiology and Chronic Health Evaluation; CI, confidence interval; ICU, intensive care unit; OR, odds ratio.

**Table 4:** SOFA score and age-adjusted multivariate analysis for risk factors for hospital mortality

	OR	95% CI	*P *value
ICU-acquired infection	2.7	1.34–5.40	0.005
Malignancy or immunosuppressive medication	2.4	1.25–4.68	0.009
Community-acquired pneumonia	3.9	1.9–7.9	<0.001

-2 Log likelihood 255,837, *P *(Hosmer and Lemeshow Test) = 0.660. CI, confidence interval; ICU, intensive care unit; LOS, length of stay; OR, odds ratio; SOFA, Sequential Organ Failure Assessment.

**Table 5:** Outcome data according to infection status on admission and ICU-acquired infection

	No infection on admission, no ICU-acquired infection (N = 49)	No infection on admission, but ICU-acquired infection (N = 35)	Infection on admission, no ICU-acquired infection (N = 206)	Infection on admission, also ICU-acquired infection (N = 45)	*P *value
Total TISS score^a^	170 (130–274)	324 (255–510)	197 (136–278)	668 (397–1,013)	<0.001
LOS in ICU (days)	3 (2.2–5.3)	7.6 (5.5–11.2)	4 (2.8–5.9)	14 (7–21.6)	<0.001
LOS in hospital (days)	17 (8–25.5)	18 (12–33)	16 (9–27)	31 (24–43)	<0.001
ICU mortality	1 (2.0)	3 (8.6)	12 (5.8)	4 (8.9)	0.43
Total hospital mortality	3 (6.1)	9 (25.7)	35 (17)	16 (35.6)	0.002
Observed/predicted mortality^b^	0.15 (0.03–0.44)	0.75 (0.34–1.42)	0.35 (0.25–0.49)	0.70 (0.40–1.13)	

Values are presented as median (with 25th to 75th percentiles in parentheses), as the number (percentage) of patients or as ratio (with 95% CI). ^a^Therapeutic intensity score during the whole ICU stay. ^b^Calculated according to APACHE II score on admission. ICU, intensive care unit; LOS, length of stay; TISS, Therapeutic Intensity Scoring System.
